# Limited supply of face masks for use in healthcare settings

**DOI:** 10.1111/irv.12817

**Published:** 2020-10-08

**Authors:** Pathum Sookaromdee, Viroj Wiwanitkit

**Affiliations:** ^1^ Private Academic Consultant Bangkok Thailand; ^2^ Dr DY Patil University Pune India

Dear Editor,

We read with interest the report on “COVID‐19: Face Masks and Human‐to‐human Transmission.”^1^ Liu et al noted that “*It is advised that you bring 1‐2 extra face masks and wear a new face mask immediately if the old one is deformed or contaminated*.”^1^ In fact, face mask is a basic tool for source control of respiratory droplets from infected individuals. An important question is on the ability of surgical face masks to prevent infection in healthcare workers. The type and quality of mask is an important determinant. In some infectious diseases, virus droplet can be very small and wearing of face mask provides limited protection,^2^ although it is currently unclear whether COVID‐19 can be transmitted by small droplets.

Regarding the case described by Liu et al, face masks might be a factor that helped prevent transmission in the community. However, surgical face masks are in short supply worldwide. In healthcare settings, face masks are often used but there are also other factors for risk assessment such as immune status of medical personnel, infectivity to the patient, and clinical characteristics of the clinical environment. The patient contact period is also another important factor determining the risk of disease transmission.

In addition to protective device, additional procedures such as rearrangement of clinical practice unit for increasing space and promoting ventilation are required.

We appreciate the recommendations by Liu et al We would like to share ideas and experience from our country, Thailand, the second country with confirmed COVID‐19 cases.^3^ We have limited supply of face masks for healthcare workers. The medical personnel sometimes have to reuse face mask for many days, and this could increase the risk of infection. It is difficult to follow the recommendations on proper face mask use because of limited supply. The same problem might exist in many low‐ and middle‐income countries worldwide.

## CONFLICT OF INTEREST

None.

## AUTHOR CONTRIBUTION

**pahum sookaromdee:** Conceptualization (equal); Data curation (equal); Formal analysis (equal); Funding acquisition (equal); Investigation (equal); Methodology (equal); Resources (equal); Software (equal); Supervision (equal); Validation (equal); Visualization (equal); Writing‐original draft (equal); Writing‐review & editing (equal). **viroj wiwanitkit:** Data curation (equal); Formal analysis (equal); Funding acquisition (equal); Investigation (equal); Methodology (equal); Project administration (equal); Resources (equal); Software (equal); Supervision (equal); Validation (equal); Visualization (equal); Writing‐original draft (equal); Writing‐review & editing (equal).
